# Coupled microbial bloom and oxygenation decline recorded by magnetofossils during the Palaeocene–Eocene Thermal Maximum

**DOI:** 10.1038/s41467-018-06472-y

**Published:** 2018-10-01

**Authors:** Liao Chang, Richard J. Harrison, Fan Zeng, Thomas A. Berndt, Andrew P. Roberts, David Heslop, Xiang Zhao

**Affiliations:** 1 Laboratory of Orogenic Belts and Crustal Evolution, School of Earth and Space Sciences, Peking University, 100871 Beijing, China; 2 Laboratory for Marine Geology, Qingdao National Laboratory for Marine Science and Technology, 266071 Qingdao, China; 3 Institute of Ocean Research, Peking University, 100871 Beijing, China; 4 Department of Earth Sciences, University of Cambridge, Cambridge, CB2 3EQ UK; 5 Research School of Earth Sciences, Australian National University, Canberra, ACT 2601 Australia

## Abstract

Understanding marine environmental change and associated biological turnover across the Palaeocene–Eocene Thermal Maximum (PETM; ~56 Ma)—the most pronounced Cenozoic short-term global warming event—is important because of the potential role of the ocean in atmospheric CO_2_ drawdown, yet proxies for tracing marine productivity and oxygenation across the PETM are limited and results remain controversial. Here we show that a high-resolution record of South Atlantic Ocean bottom water oxygenation can be extracted from exceptionally preserved magnetofossils—the bioinorganic magnetite nanocrystals produced by magnetotactic bacteria (MTB) using a new multiscale environmental magnetic approach. Our results suggest that a transient MTB bloom occurred due to increased nutrient supply. Bottom water oxygenation decreased gradually from the onset to the peak PETM. These observations provide a record of microbial response to the PETM and establish the value of magnetofossils as palaeoenvironmental indicators.

## Abstract (web summary)

Understanding the response of marine productivity and CO_2_ drawdown to past warming events can provide important insights into the future. Here, the authors use bacterial magnetite nanoparticle fossils to reconstruct nutrient supply and marine deoxygenation during the Palaeocene–Eocene Thermal Maximum.

## Introduction

The Palaeocene–Eocene Thermal Maximum (PETM; ~56 Ma) represents a global warming event with the highest rate of Cenozoic temperature rise and major carbon cycle perturbation^1^ that is considered an analogue for understanding anthropogenic global warming, although probably with a slower carbon release rate^2,3^. The PETM was characterized by, among other things, rapid changes in marine biogeochemistry^4^, benthic foraminiferal extinction^5^ and changes in deep-sea circulation^6,7^. Assessing abrupt marine environmental change, associated biotic responses and CO_2_ uptake across the PETM requires an understanding of key oceanic conditions, such as productivity and bottom water oxygenation. However, temporal and spatial palaeoceanographic reconstructions of productivity and deep-sea O_2_ concentrations from geochemical and biotic proxies are limited and provide contradictory results^8–13^. This limits palaeoenvironmental reconstruction across the PETM and highlights the need for new independent proxies. The impact of PETM warming and environmental turnover on marine microbial communities has also been elusive, due to scarce microbial fossil preservation.

Magnetofossils, a term coined by Chang and Kirschvink^14^, are an important type of microbial fossil, which hold promise as recorders of palaeoenvironmental conditions across the PETM. Magnetofossils are the fossil remains of magnetite (Fe_3_O_4_) or greigite (Fe_3_S_4_) magnetosome crystals that were originally biomineralized by magnetotactic bacteria (MTB)^14–16^. MTB often live in chemically stratified aquatic environments near the water–sediment interface^15^, although magnetofossils have also been identified in marine environments without redox gradients where MTB may live within surface sediments^17,18^. MTB are a diverse group of aquatic prokaryotes that biomineralize membrane-enclosed magnetic nanoparticles that are arranged in chains as microscopic compasses that cause passive alignment of the bacteria in the geomagnetic field, allowing them to migrate efficiently to their preferred oxygen concentration^15^. They are the most primitive organisms that mineralize iron minerals with well-constrained size and shape under a high degree of genetic control^15,16^. MTB are distributed widely in natural environments, and magnetosome crystals can be buried and preserved as magnetofossils that record palaeo-productivity^19–21^, glacial–interglacial cycles^22^, long-term palaeo-redox conditions^22–24^ and other palaeoenvironmental information^25,26^.

Kent et al.^27^ initially observed anomalous magnetic properties within the PETM interval from continental shelf sediments in New Jersey, from which they proposed a comet impact trigger hypothesis for the PETM. Such unusual magnetic properties within the PETM were demonstrated later to be due to magnetofossil occurrence^28,29^. Discovery of novel large magnetofossil crystals from electron microscope analyses of magnetic mineral extracts^30,31^, and identifications of marine magnetofossil records of Eocene hyperthermal events from globally distributed ocean drilling cores and continental outcrops^32–34^, highlight the usefulness of magnetofossil records to understand the PETM and other ancient warming events.

Direct analysis of magnetofossil morphology—the primary visible characteristic of magnetofossils—in sedimentary records using transmission electron microscope (TEM) observations—has been used widely to identify and characterize magnetofossils since the 1980s^35–39^. However, statistical analysis of magnetofossil morphologies using a large number of crystals is relatively rare. In addition, interpretation of magnetofossil records is hindered by the lack of suitable tools to link directly the magnetofossil morphology to corresponding magnetic properties.

**Fig. 1:**
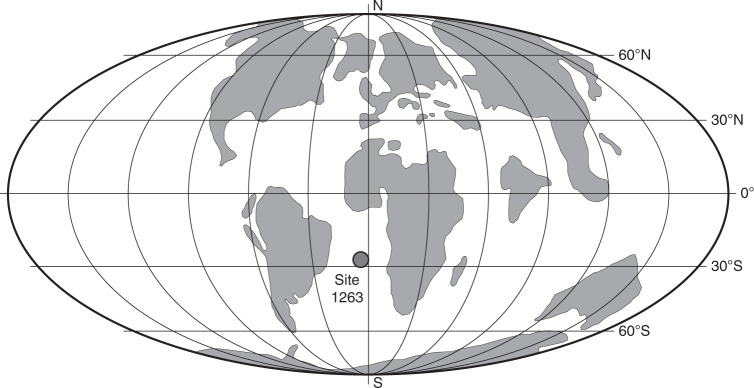
Location of ODP Site 1263 during the Late Palaeocene. The core (current latitude: 28°32′S; longitude: 02°47′E) is on Walvis Ridge, southeastern Atlantic Ocean. Plate tectonic reconstruction is from the Ocean Drilling Stratigraphic Network (ODSN) database at http://www.odsn.de

Here, we develop a new multiscale approach based on TEM observations, magnetic properties and micromagnetic simulations to characterize trace magnetofossil concentrations preserved within PETM sediments. We use magnetofossil abundance and morphology signatures, from direct TEM imaging and bulk magnetic property measurements, to trace palaeo-environmental conditions. Three-dimensional micromagnetic models were built to simulate magnetofossil magnetic properties and to test the robustness of magnetic proxies. Pelagic PETM sediments from Walvis Ridge, South Atlantic Ocean Drilling Program (ODP) Hole 1263C (refs. ^4,40,41^) were analysed here (Fig. 1 and Supplementary Table 1) to explore the microbial response and to reconstruct bottom water oxygenation through the PETM.

## Results

### Magnetic properties across the PETM at Site 1263

**Fig. 2:**
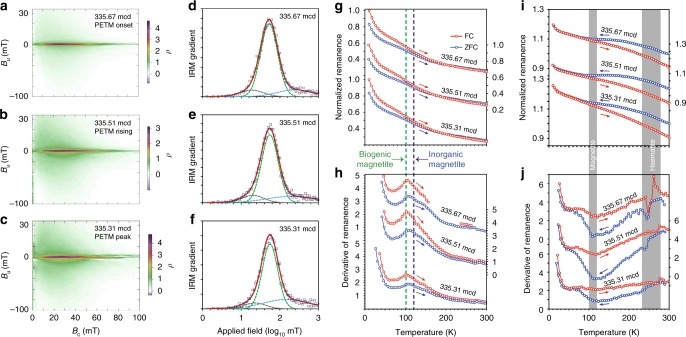
Magnetic data for three typical PETM samples from ODP Hole 1263C. The three samples are section 14H-2A, 146–147 cm interval, at: 335.67 metres composite depth (mcd; PETM onset), section 14H-2A, 130–131 cm interval, at 335.51 mcd (rising PETM), and section 14H-2A, 110–111 cm interval, at 335.31 mcd (PETM peak). **a**–**c** FORC diagrams, **d**–**f** IRM coercivity components, **g** low-temperature SIRM warming and **h** corresponding derivative curves, and **i** LTC of a room temperature SIRM and **j** corresponding derivative curves. In **d**–**f**, squares indicate measured data, thick red lines are fitted total coercivity spectra, and dashed lines represent fitted components: green, biogenic magnetite; grey, detrital soft magnetic mineral assemblage; and blue, detrital hard magnetic mineral assemblage. In **g**, **h**, SIRM warming curves are normalized to the magnetization at 10 K in FC curves. Vertical dashed lines mark temperatures of 100 and 120 K (i.e., the Verwey transition temperatures for biogenic and detrital magnetite, respectively^46^. In **i**, **j**, LTC curves are normalized to the initial SIRM at room temperature. Grey bars indicate temperature intervals for phase transitions in magnetite and haematite

The PETM interval at Site 1263 is a grey-brown clay layer, whereas before and after the PETM the sediment is a carbonate-rich ooze^4^. Magnetic measurements were made on samples across the PETM interval from Hole 1263C, core 14H, and sections 2A and CC (Supplementary Table 1), which provide strong evidence of magnetofossil occurrences within the studied PETM interval. First-order reversal curve (FORC) diagrams^42^ (Fig. 2a–c) contain a major central ridge component along *B*_u_ = 0, which is an indicator of magnetofossils in sediments^43^. The weaker part of the FORC distribution with large vertical spread is probably associated with contributions from collapsed magnetosome chains and detrital magnetic minerals^43,44^. Coercivity decomposition of isothermal remanent magnetization (IRM) acquisition curves (Fig. 2d–f) indicates a dominant component with small dispersion parameter (DP) values^45^. Low-temperature warming of a saturation IRM (SIRM) after zero-field-cooled (ZFC) and field-cooled (FC) treatments indicates a distinct peak at ~100 K (Fig. 2g, h) due to the Verwey transition (*T*_V_) of biogenic magnetite^46^. The *T*_V_ signature of biogenic magnetite is also expressed in the low-temperature cycling (LTC) of a room temperature SIRM (Fig. 2i, j). Haematite is detected by the Morin transition (*T*_M_)^47^ in LTC curves (Fig. 2i, j). Carbonate oozes above and below the PETM are magnetically weak and are dominated by paramagnetism.

**Fig. 3:**
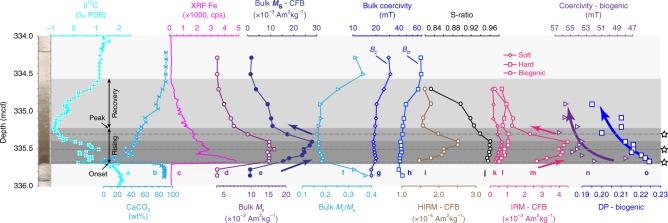
Down-core magnetic profiles for 16 discrete samples across the PETM for ODP Hole 1263C. **a**–**c** Published bulk sediment carbon isotope, carbonate content, and Fe concentration (X-ray fluorescence measurements)^4^. **d**, **e** Bulk *M*_s_, **f** *M*_rs_/*M*_s_, **g** bulk coercivity, **h** bulk coercivity of remanence, **i** hard IRM plotted on a carbonate-free basis (HIRM-CFB), **j** S-ratio, **k**–**m** IRM-CFB coercivity components, **n** fitted *B*_1/2_ and **o** DP values for the biogenic magnetite component from IRM curves. Left: Core photo of the PETM interval in Hole 1263C^40^. Horizontal dashed lines and stars indicate positions of the three samples with magnetic properties presented in Fig. 2, and detailed TEM observations presented in Fig. 4. Grey bars mark different stages across the PETM. Arrows in **e**, **m**, **n**, **o** are visual guides for data trends

Magnetic parameters are used to trace changing magnetofossil signatures across the PETM (Fig. 3). Consistent with previous studies on other PETM sections^19,28,29,32^, biogenic magnetite represents a major magnetic mineral component for samples from Site 1263, particularly from the early PETM stage. For example, quantitative IRM decomposition analysis indicates that the magnetofossil component contributes ~76% to the total remanent magnetization for the PETM onset sample (section 14H-2A, 146–147 cm interval; 335.67 metres composite depth (mcd)). The FORC diagram for this sample (Fig. 2a) contains a prominent central ridge signal due to magnetofossils. From the PETM rising to recovery stage, there is a gradually increasing contribution from the magnetically harder haematite assemblage, as indicated by the carbonate-free hard IRM (HIRM) parameter (Fig. 3i). For example, for the peak PETM sample (section 14H-2A, 110–111 cm interval; 335.31 mcd), haematite contributes 28% to the total SIRM, compared to 15% for the PETM onset sample. Magnetic concentration-dependent parameters, such as carbonate-free saturation magnetization (*M*_s_; Fig. 3e), broadly reflect magnetofossil content changes. We also use IRM decomposition analysis to separate the magnetofossil contribution from other magnetic mineral sources to assess magnetofossil abundance changes through the PETM. An abrupt magnetofossil increase precisely at the PETM onset is followed by a gradually increasing magnetofossil concentration before peaking slightly prior to the δ^13^C peak (Fig. 3a, e, m). Magnetofossil abundance during the PETM recovery is much lower than during the warming stage. During the recovery, the relative abundance of magnetically hard minerals, that is, haematite, with respect to biogenic magnetite increases markedly, as reflected in the decreased S-ratio (Fig. 3j). Such magnetic hardening during PETM recovery is also reflected in the shape of the IRM curves and bulk coercivity values (Fig. 3g, h). Fitted parameters for magnetofossil IRM components indicate a gradual increase in *B*_1/2_ (magnetofossil coercivity) and gradually decreased DP values during PETM warming (Fig. 3m, n). Magnetofossil coercivity values remain high with low DP values during the recovery.

### Crystal morphologies of magnetofossils

**Fig. 4:**
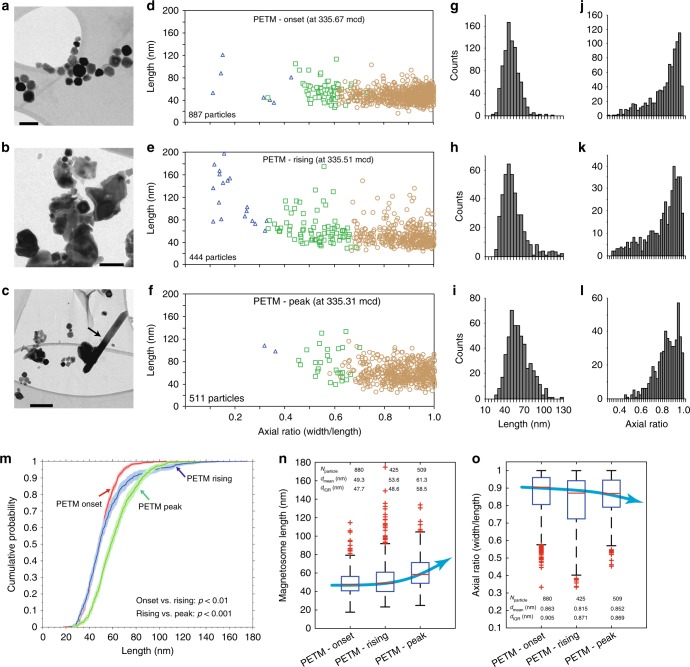
TEM observations and statistical analyses of magnetofossil morphologies for three typical PETM samples from ODP Hole 1263C. **a**–**c** Bright-field TEM images. Scale bars in **a** and **b**, **c** are 100 and 200 nm, respectively. The arrow in **c** indicates a needle-shaped giant magnetofossil crystal. **d**–**f** Length–axial ratio (width/length) diagrams for magnetofossil crystals measured from the TEM images. Magnetofossil morphologies are categorized into three main groups: isotropic or nearly isotropic octahedral and cubo-octahedral crystals (orange circles), elongated crystals and prisms (green squares), and bullet-shaped (blue triangles) crystals. The number of counted magnetofossil crystals is shown. Histograms of **g**–**i** length and **j**–**l** axial ratio for magnetofossils excluding bullet-shaped crystals. **m** Cumulative probability of length of magnetofossils excluding bullet-shaped crystals for the three samples with Kolmogorov–Smirnov test results. Box-whisker plots of **n** length and **o** axial ratio of magnetofossils excluding bullet-shaped crystals. The samples are the same as in Fig. 2. (**a**, **d**, **g**, **j**), (**b**, **e**, **h**, **k**) and (**c**, **f**, **i**, **l**) are for PETM onset, rising and peak samples, respectively

Three typical samples, selected from the onset (section 14H-2A, 146–147 cm interval; 335.57 mcd), rising (section 14H-2A, 130–131 cm interval; 335.51 mcd) and peak (section 14H-2A, 110–111 cm interval; 335.31 mcd) PETM stages, were subjected to detailed TEM and scanning electron microscope (SEM) observations, which provide direct evidence of magnetofossil occurrence (Fig. 4a–c). Variable morphologies, mostly cubo-octahedra with different elongations, are observed. Bullet-shaped crystals are rare. For example, observed bullet-shaped crystals represent only 0.8, 4, and 0.4% of the total magnetofossil crystal counts for the onset, rising, and peak PETM samples, respectively (Fig. 4d–f). The observed magnetite nanocrystals are similar to those of typical magnetosomes reported from MTB^15,16^, with well-defined crystal morphologies with sharp crystal boundaries, and narrow size distributions that fall well within the theoretical stable single-domain (SD) size range for magnetite^48^. Our micromagnetic modelling below also indicates that if the observed magnetite crystals are isolated, the calculated coercivity is far too low compared to experimentally determined coercivities. This indicates that significant amounts of the magnetite crystals must be preserved in chain structures within the PETM samples, the strong anisotropy of which increases the coercivity. The observed crystal morphologies, size distributions, and microstructures contrast significantly with those expected from detrital magnetic minerals that often do not have well-defined crystal shapes, small size distributions, and a lack of chain structures. Therefore, our TEM analyses, magnetic measurements, and simulations indicate consistently that magnetic nanoparticles within the PETM samples from Site 1263 are magnetofossils, and that other origins, such as comet-induced debris^27^ and pyrogenesis^49^, are improbable. Magnetofossils are also evident in high-resolution SEM observations (Supplementary Fig. 1). In addition to the observed magnetofossil nanocrystals, TEM observations reveal the presence of giant magnetite needles within the peak PETM (Fig. 4c and Supplementary Fig. 2), with similar morphologies to those reported previously within PETM sediments^30,31^. No spindles, spear-head shaped grains^30^ or giant bullet-shaped magnetite crystals^31^ were found within the studied PETM sediments from Site 1263. Giant magnetite needles were observed only within samples from the peak PETM stage, and not within the onset and rising PETM stages.

It has been suggested that magnetofossil morphology can be an indicator of sedimentary palaeo-oxygenation state^21,22,24,25^. A comprehensive linkage between magnetofossil morphology and palaeo-oxygen levels does not exist because of the need for large magnetofossil morphology datasets to demonstrate the relationship. We imaged, counted, and analysed statistically >1700 magnetofossil crystals from three PETM stages (Fig. 4). Such large datasets enable assessment of magnetofossil size and morphology distributions. We divide magnetofossil morphologies into three groups: octahedra and cubo-octahedra, elongated crystals and prisms, and bullet-shaped crystals (Fig. 4d–f). Histograms of magnetofossil lengths (Fig. 4g–i) excluding bullet-shaped crystals indicate nearly log-normal distributions that are distinct for the three analysed samples. A Kolmogorov–Smirnov test of the cumulative probability confirms that the three distributions are statistically different (Fig. 4m). The axial ratio (Fig. 4j–l) also has different distributions. Box-whisker plots of length (Fig. 4n) and axial ratio (Fig. 4o) of magnetofossil crystals excluding bullets indicate clear trends: magnetofossil length increases, and axial ratio decreases, from the onset to the peak PETM.

### Micromagnetic simulation of magnetofossil properties

**Fig. 5:**
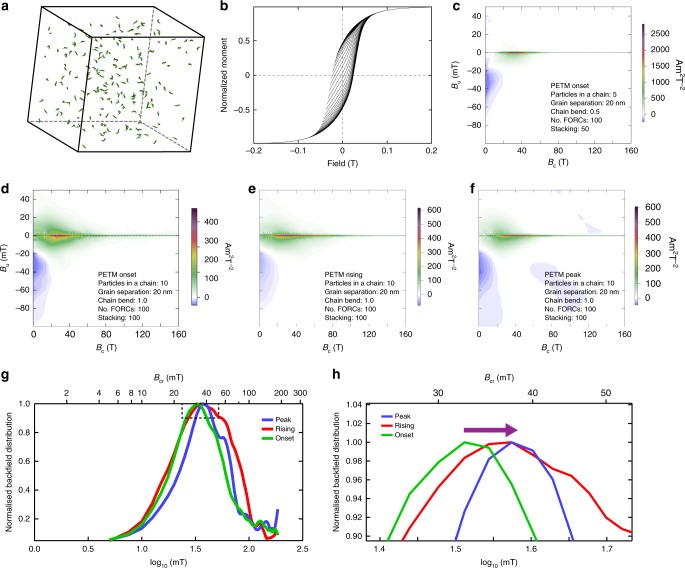
Micromagnetic simulations of magnetofossils for samples from ODP Hole 1263C. **a**–**c** Micromagnetic simulation of FORC diagrams for PETM onset sample: **a** geometry, **b** simulated FORCs, and **c** processed FORC diagram. For input models, particle size distributions of biogenic magnetite are from TEM observations (Fig. 4). The magnetosome chain configurations modelled in **c** are: 5 crystals in a chain, 60 chains, a chain bending factor of 0.5, a grain separation of 20 nm, and randomly oriented chains with packing fraction of 0.001 (ref. ^44^). **d**–**f** Micromagnetic simulation of FORC diagrams for the three PETM samples using model parameters: 10 crystals in a chain, a chain bending factor of 1.0 (collapsed chains) and a grain separation of 20 nm. The three simulations represent the samples for which experimental results are presented in Figs. 2 and 4. For all FORC simulations, we modelled 100 FORCs with *B*_c _= 160 mT, *B*_u _= 60 mT, and averaged 100 identical FORC simulations. Particles were selected randomly again from the TEM observations for each FORC simulation. FORC diagrams were processed using FORCinel^67^ with VARIFORC smoothing parameters:^68^ {*s*_c0_, *s*_c1_, *s*_b0_, *s*_b1_, *λ*_c_, *λ*_b_} = {7, 7, 2, 7, 0.1, 0.1}. **g**, **h** Back field coercivity distributions extracted from the simulated FORC diagrams in **d**–**f** for the three PETM samples. **h** Data from the enlarged dashed box in **g**; the arrow in **h** indicates the data trend

To understand the link between magnetofossil size distribution and bulk magnetic properties, we built three-dimensional micromagnetic models (Supplementary Fig. 3) using real magnetofossil size distributions from TEM observations of magnetic mineral extracts of the onset, rising and peak PETM samples. Such a model enables simulation of the magnetic properties of magnetofossils from different PETM stages (Fig. 5). Varying biogenic chain architectures were modelled, that is, the number and separation of particles in a chain, chain bending and chain packing factors^44^. Our simulations indicate that all of these factors can affect the measured magnetic properties (Fig. 5 and Supplementary Fig. 3). For example, coercivity increases with the number of particles in a chain, whereas it decreases with increased degree of chain bending and packing, as has been observed experimentally on collapsed bacterial chains using ultrasonic disruption^50,51^. Although exact chain arrangements in sediments are unknown, our simulations indicate the same trend as measured magnetic properties (i.e., magnetofossil coercivity increases from the onset to peak PETM), when using the same modelling parameters but with different particle size distributions. For example, calculated coercivity increases from the PETM onset to the peak (Fig. 5), as observed in our experimental data (Figs. 2 and 3). Our simulations link directly biogenic magnetite size distributions and bulk magnetic properties, which justifies use of magnetic proxies (Fig. 3m, n), such as the experimentally determined coercivity of the biogenic magnetite (Fig. 3), to reflect changes in magnetofossil size and morphology.

## Discussion

MTB are sensitive to redox changes and are found mostly near the oxic–anoxic interface in aquatic habitats^15^. Microaerobic conditions are argued to be crucial for biomineralization in MTB^52^, as has been demonstrated by a strong link between magnetosome morphology and oxygen content in laboratory-controlled MTB cultures^16,53^ and magnetofossil changes during glacial–interglacial cycles^21,22^. For example, high oxygen concentrations suppress magnetite biomineralization so that smaller, and aberrantly shaped crystals form^15,16,53^. Our magnetic property and statistical analysis of magnetofossil morphologies at Site 1263 indicate a consistent trend: the average size and elongation of magnetosome crystals increased from the onset to the PETM peak. This MTB response reflects a gradual decrease in deep-sea oxygenation. The ratio of bullet-shaped magnetofossils to the other morphologies has been used as an indicator of relative organic carbon flux^54^. The number of observed bullet-shaped magnetofossils is small, which makes it difficult to draw a definite conclusion for Site 1263. Nevertheless, a decline in deep water oxygenation during the PETM at Site 1263 is clearly reflected in our magnetofossil assemblage record, which is consistent with geochemical proxies^13^, benthic foraminiferal evidence^55–57^ and climate-carbon cycle modelling^58^.

Magnetofossils were not detected within pre-PETM carbonate oozes. For example, samples from just below the PETM onset in Hole 1263C, core 14H-CC, are dominated by paramagnetism with negligible ferrimagnetic mineral contents. A sudden magnetofossil abundance increase at the PETM onset was followed by a gradual increase to a peak slightly below the δ^13^C peak (Fig. 3). The magnetofossil abundance increase reflects an increased nutrient supply for MTB biomineralization over the PETM and during the warming stage. A marked magnetofossil decline during the PETM recovery stage, as reflected in the intensity of the magnetofossil IRM component (Fig. 3m), then reflects decreasing nutrient supply.

Roberts et al.^20^ demonstrated that MTB production in marine environments is limited by nutrient supply, mainly through iron availability and organic carbon delivery that is necessary for MTB metabolism, which can be linked ultimately to marine primary productivity. The observed PETM MTB bloom could indicate either a productivity rise (through burial of more organic carbon) or increased bioavailable iron. Increased productivity could be due to intensified continental weathering and nutrient run-off that reached the studied pelagic South Atlantic site, as has been suggested to explain North Atlantic^19^ and Southern Ocean^32^ PETM magnetofossil records. Continentally derived biomarkers are present within the PETM sediments at Site 1263, but they have low concentrations^59,60^. Higher organic carbon delivery to the seafloor at Site 1263 is also inconsistent with benthic foraminiferal evidence^55–57^. Moreover, increased productivity is not expected in open-ocean environments, where modelling predicts that thermally enhanced stratification will cause less efficient biological pumping and decreased productivity associated with warmer and more nutrient-depleted conditions during periods of global warming^58,61,62^.

The second, and more likely, cause for the PETM MTB bloom at Site 1263 is increased iron bioavailability. Magnetosome production by MTB and their geological preservation requires a fine balance between having sufficient organic carbon flux to cause diagenetic release of reactive iron for magnetosome biomineralization, while at the same time not having so much organic carbon that sedimentary sulphidic diagenetic conditions are produced, which are corrosive to magnetite^20,63^. SEM observations reveal features associated with mild diagenetic magnetic mineral corrosion in more reducing environments at Site 1263 (Supplementary Fig. 1), which is consistent with lower PETM O_2_ levels. Overall, magnetofossil preservation and evidence for only mild magnetic mineral diagenesis indicate that the required balance between organic carbon export to the seafloor and lack of reductive diagenesis has been maintained through the PETM at Site 1263. Decreased oxygenation levels during the PETM at Site 1263 could stimulate, through reductive diagenesis, release of more iron for magnetite biomineralization. Likewise, lack of increased productivity, as indicated by benthic foraminiferal evidence^55–57^ and modelling predictions^58^, during the PETM at Site 1263 would have prevented sulphidic diagenetic conditions from being reached. This interpretation, with decreased oxygenation and no productivity rise, is consistent with our magnetofossil morphological data, and would have helped to ensure that the fine diagenetic balance between organic carbon export to the seafloor and lack of reductive diagenesis required for magnetofossil preservation was maintained through the PETM at Site 1263.

In summary, our magnetofossil record and other magnetic evidence consistently indicate a deep-sea O_2_ decline during the PETM at Site 1263. Global warming would have contributed to driving lower surface and deep ocean oxygen solubility, which would also have caused low oxygen contents during the PETM^58^. Warming can also produce more stratified conditions, with reduced ocean overturning circulation, to make deep-sea circulation sluggish^13^. Low oxygenation can also be caused by increased regional oxygen utilization due to enhanced carbon influx in the deep sea, enhanced productivity^11^ and through methane oxidation released from clathrates^64^. At Site 1263, bottom water warmed considerably more than the global average^59^, possibly due to circulation changes^65,66^. It is possible that oxygenation and productivity may have been decoupled, such that the origin of the O_2_ decease is uncertain. However, our magnetofossil record of decreased oxygenation provides important constrains for understanding environmental change and biological turnover during the PETM^56,57^.

## Methods

### Magnetic extractions and TEM and SEM observations

TEM samples were prepared following ref ^31^. About 1 cm^3^ of untreated sediment was mixed with distilled water, and was placed in an ultrasonic bath for ~5 min. Magnetic extraction was performed with a Frantz isodynamic magnetic separator and a long glass tube with a stopcock at the base. For extraction, the tube was filled with distilled water and was placed between the electromagnet pole pieces, which was then switched on and sediment solution was added slowly into the tube from the top. Magnetic minerals, including magnetofossil crystals, were trapped onto the tube walls, while non-magnetic materials were deposited at the bottom of the tube. This procedure was repeated to increase the amount of magnetic material on the tube wall. Finally, magnetic minerals were washed off the tube wall into a container and were purified using a strong rare-earth magnet. A TEM grid was floated on top of the solution containing the extracted magnetic materials with a rare-earth magnet suspended above the TEM grid. Magnetic extracts were viewed and analysed using a Philips CM300 TEM operated at 300 kV at the Australian National University (ANU). SEM observations were made using a Carl Zeiss UltraPlus analytical field emission SEM with an INCA ENERGY 450 energy-dispersive X-ray spectroscopy system at ANU.

### Magnetic measurements

Hysteresis, IRM acquisition curves, and FORC measurements were made using a Princeton Measurements Corporation vibrating sample magnetometer at ANU. IRM curves contain 80 data points that were measured at logarithmically spaced field steps, which were decomposed following ref ^45^. FORC measurements were made with a field step of 0.6 mT with averaging times of 150–400 ms. FORC data were processed using the FORCinel package^67^ with the VARIFORC protocol^68^. The magnetic S-ratio proxy was calculated as: S-ratio = –IRM_−0.3 T_/IRM_1 T_. The magnetic HIRM proxy was calculated as: HIRM = (IRM_1 T_ – IRM_−0.3 T_)/2. Low-temperature magnetic properties were measured with a Quantum Design Magnetic Property Measurement System (MPMS; model XL7) at ANU. For low-temperature SIRM warming in ZFC and FC conditions, samples were cooled to 10 K in either zero field or a 5 T field. At 10 K, a 5 T field was applied (for 1 min), then switched off, and the MPMS magnet was reset. ZFC and FC curves were measured during warming in zero field. For LTC of a room temperature SIRM, remanence was measured from room temperature to 10 K and back to room temperature in zero field at a heating rate of 5 K per min.

### Micromagnetic simulations

Micromagnetic calculations are based on interacting ensembles of stable SD particles. Numerical integration of the Landau–Lifshitz–Gilbert (LLG) equation was used to calculate the equilibrium magnetization of the ensemble, considering dipole–dipole magnetostatic interactions at each step:

1 $$\frac{{{\mathrm{d}}{\boldsymbol{M}}}}{{{\mathrm{d}}t}} = - \frac{\gamma }{{1 + \alpha ^2}}{\boldsymbol{M}} \times {\mathbf{H}}_{{\mathrm{eff}}} + \frac{{\alpha \gamma }}{{\left( {1 + \alpha ^2} \right)M_{\mathrm{s}}}}{\boldsymbol{M}} \times \left( {{\boldsymbol{M}} \times {\mathbf{H}}_{{\mathrm{eff}}}} \right),$$

where ${\mathbf{H}}_{{\mathrm{eff}}} = - \frac{1}{{\mu _0}}\frac{{{\mathrm{d}}E_{{\mathrm{Total}}}}}{{{\mathrm{d}}{\boldsymbol{M}}}}$, *γ* is the gyromagnetic frequency and *α* is a damping parameter. Direct integration of the LLG equation in the time domain was not used in our micromagnetic calculations, but an approximate method was adopted, whereby the magnetic configuration is relaxed iteratively by placing each magnetization vector close to the effective field vector throughout the ensemble^44^. Such a micromagnetic approach is appropriate for modelling biogenic magnetite assemblages with dominant SD particles^44^. Compared to full micromagnetic simulation, this simplified method is computationally relatively rapid, which makes it efficient to compute results for large number of particles to understand natural samples. Micromagnetic calculations were made using a modified version of FORCulator^44^ by considering particle size distributions determined experimentally from TEM imaging. To build magnetofossil ensembles, magnetite grains were selected randomly from the particle distributions directly in our TEM observations, which were then assembled into different architectures. The coercivity for elongated magnetite particles is given by:

2 $$B_{\mathrm{c}} = \Delta N \cdot M_{\mathrm{s}},$$

where Δ*N* is the difference between the self-demagnetizing factors along the particle width and length and *M*_s_ is the saturation magnetization for magnetite at room temperature (*M*_s_ = 480 kA m^−1^)^69^. Δ*N* is calculated using the analytical formula for an elongated parallelepiped of square-cross section from ref ^70^. Cubic anisotropy was not considered in our simulations. This simplification is reasonable because shape anisotropy dominates the anisotropy even when a magnetite grain is slightly elongated, for example, length/width ratio >1.05. Length/width ratios for most magnetite crystals from our TEM observations of PETM samples are well above this threshold.

### Code availability

The code for micromagnetic simulation of magnetofossils is modified from the FORCulator software and is available at: https://wserv4.esc.cam.ac.uk/nanopaleomag/.

## Electronic supplementary material

Supplementary Information

## Data Availability

Measured magnetic parameters are in Supplementary Table 1. The data that support the findings of this study are available from the corresponding author upon request.
