# Supplementary material for: Coupled microbial bloom and oxygenation decline recorded by magnetofossils during the Palaeocene–Eocene Thermal Maximum
Source: Nat Commun. 2018 Oct 1;9:4007. doi: 10.1038/s41467-018-06472-y (PMC6167317; doi:10.1038/s41467-018-06472-y)
Supplement: Supplementary file 1 — Supplementary Information [file 41467_2018_6472_MOESM1_ESM.pdf]

Coupled microbial bloom and oxygenation decline recorded by magnetofossils during the Palaeocene-Eocene Thermal Maximum

Chang et al.

Supplementary Information

Supplementary Table 1 Magnetic properties of analysed samples from ODP Site 1263

ODP Hole	Section	Interval (cm)	Depth (mbsf)	Depth (mcd)	Bulk M_s ($\times10^{-3}$ Am^2kg^{-1})	CFB Bulk M_s ($\times10^{-3}$ Am^2kg^{-1})	Bulk B_c (mT)	Bulk B_{cr} (mT)	CFB HIRM ($\times10^{-4}$ Am^2kg^{-1})	S-ratio	CFB Biogenic IRM ($\times10^{-3}$ Am^2kg^{-1})	Biogenic B_{cr} (mT)	Biogenic DP
1263C	14H2A	8-9	283.98	334.29	0.156	1.657	29.3	77.0	-	-	-	-	-
1263C	14H2A	30-31	284.20	334.51	0.130	1.285	30.8	77.8	-	-	-	-	-
1263C	14H2A	50-51	284.40	334.71	0.537	3.631	25.4	70.9	1.613	0.832	0.260	66.1	0.180
1263C	14H2A	70-71	284.60	334.91	2.111	8.688	20.3	49.3	1.784	0.896	0.937	55.6	0.195
1263C	14H2A	89-90	284.79	335.10	4.092	10.204	20.0	46.3	1.572	0.917	1.315	55.6	0.210
1263C	14H2A	99-100*	284.89	335.20	4.900	10.793	19.6	45.0	1.560	0.925	1.240	54.1	0.210
1263C	14H2A	110-111	285.00	335.31	8.933	17.179	19.2	44.2	1.975	0.938	2.287	56.4	0.200
1263C	14H2A	120-121	285.10	335.41	15.150	27.849	18.5	41.8	2.514	0.960	4.453	53.5	0.216
1263C	14H2A	125-126	285.15	335.46	15.162	26.930	18.8	42.6	2.499	0.959	4.240	53.8	0.212
1263C	14H2A	130-131*	285.20	335.51	16.602	24.308	18.8	42.7	2.122	0.960	3.930	54.3	0.216
1263C	14H2A	134-135	285.24	335.55	14.637	24.559	19.2	43.2	2.136	0.958	4.170	55.1	0.215
1263C	14H2A	138-139	285.28	335.59	15.142	23.623	19.5	41.5	2.068	0.959	4.047	53.5	0.220
1263C	14H2A	142-143	285.32	335.63	15.154	17.976	20.1	41.0	1.781	0.954	3.264	47.9	0.222
1263C	14H2A	146-147*	285.36	335.67	15.043	15.445	19.5	41.0	1.469	0.955	2.670	52.7	0.228
1263C	14HCC	7-8	285.47	335.78	0.138	1.104	17.4	39.7	-	-	-	-	-
1263C	14HCC	15-16	285.55	335.86	0.120	1.044	17.4	44.3	-	-	-	-	-

* Samples that were subjected to detailed transmission electron microscope (TEM) and scanning electron microscope (SEM) analyses, and micromagnetic simulation

- Magnetically weak samples with noisy IRM curves

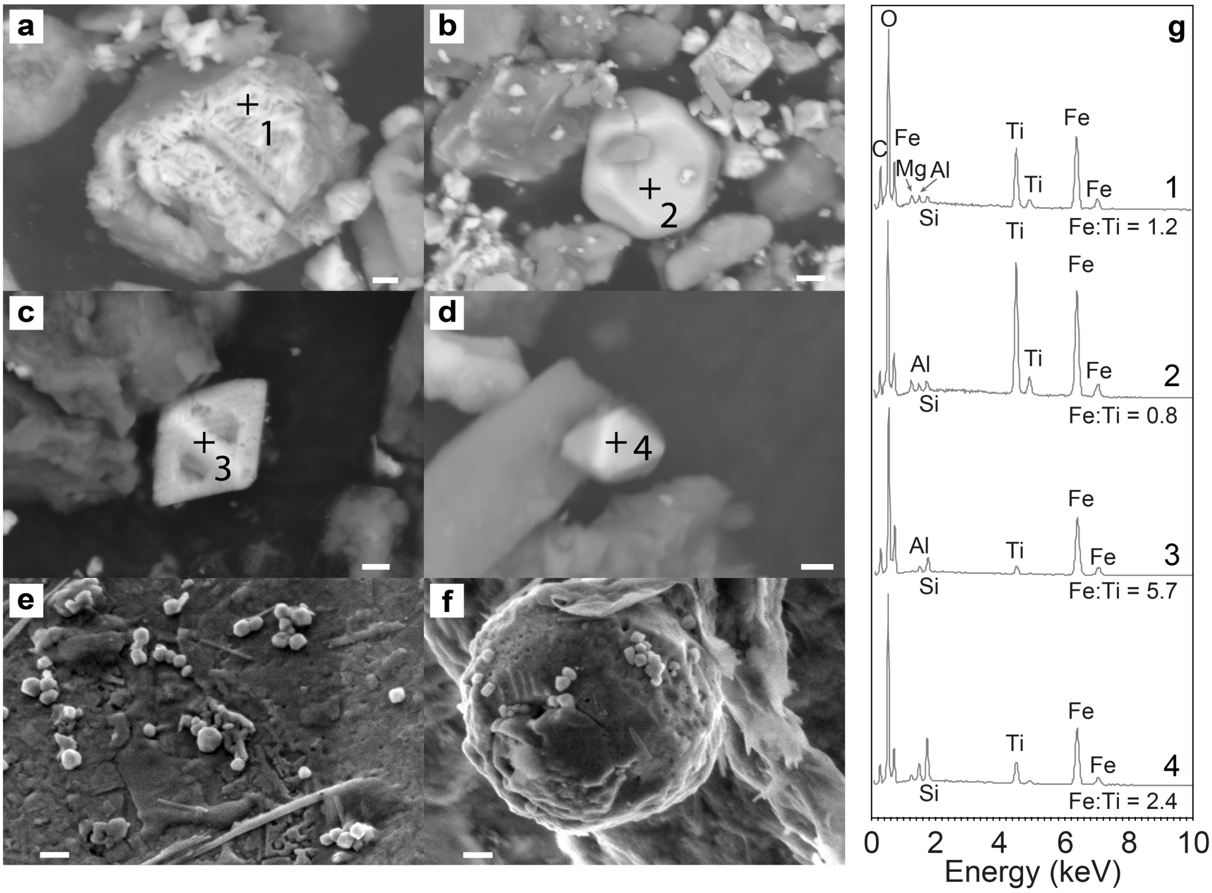

Supplementary Figure 1 SEM observations on magnetic mineral extracts of samples from ODP Site 1263. (a-f) SEM images and (g) energy-dispersive spectra (EDS) spot analyses of magnetic extracts from (a, b) the peak PETM (1263C-14-2H-110-112 cm) and (c-f) the PETM onset (1263C-14-2H-146-147 cm) at Site 1263. Scale bars in (a, c, d), (b) and (e, f) are 1 μm, 2 μm and 100 nm, respectively. SEM observations with EDS analysis reveals the presence of detrital Fe-Ti oxides (a-d) in addition to magnetofossils. Features associated with magnetic mineral dissolution[1,2] are evident in SEM observations (a), which is consistent with decreased oxygenation during PETM warming. We did not observe dissolution features associated with magnetofossils, such as magnetofossils with intact lattice fringes and dissolved internal sections, as has been observed previously[3]. High-resolution SEM images (e, f) further confirm the presence of magnetofossils within the PETM samples, which also reveal the three-dimensional shapes of magnetofossil crystals. Low-resolution SEM images (a-d) were taken at 15 kV with a sample stage of ~10-11 mm. High-resolution SEM images (e, f) were taken at 2 kV with a sample stage of 3.4 mm.

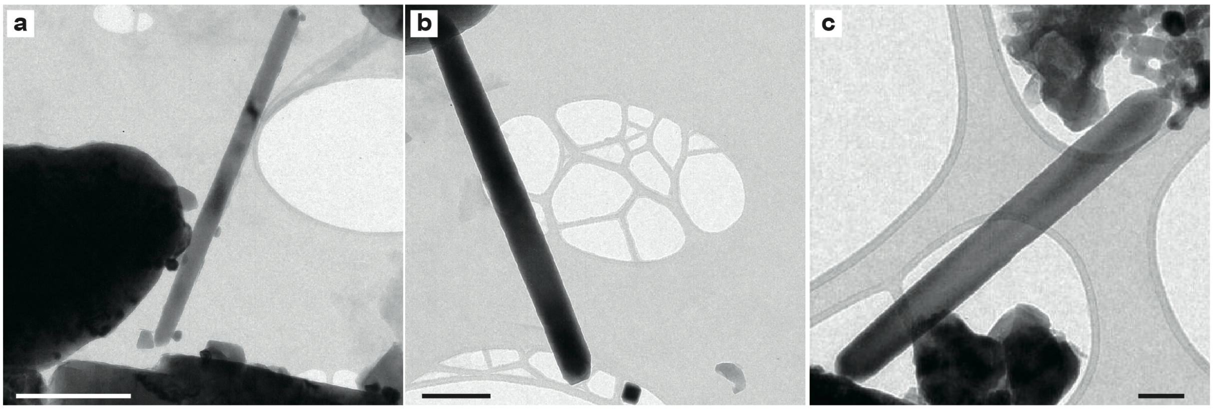

Supplementary Figure 2 TEM images of needle-shaped giant magnetofossils from the peak PETM at Site 1263 (1263C-14-2H-110-112 cm). Scale bars in (a), (b) and (c) are 500 nm, 200 nm, and 100 nm, respectively. TEM observations indicate the presence of giant needle-like magnetofossils[4,5] within the sample from the PETM peak. Magnetite needles were not observed within samples from the PETM onset and rising.

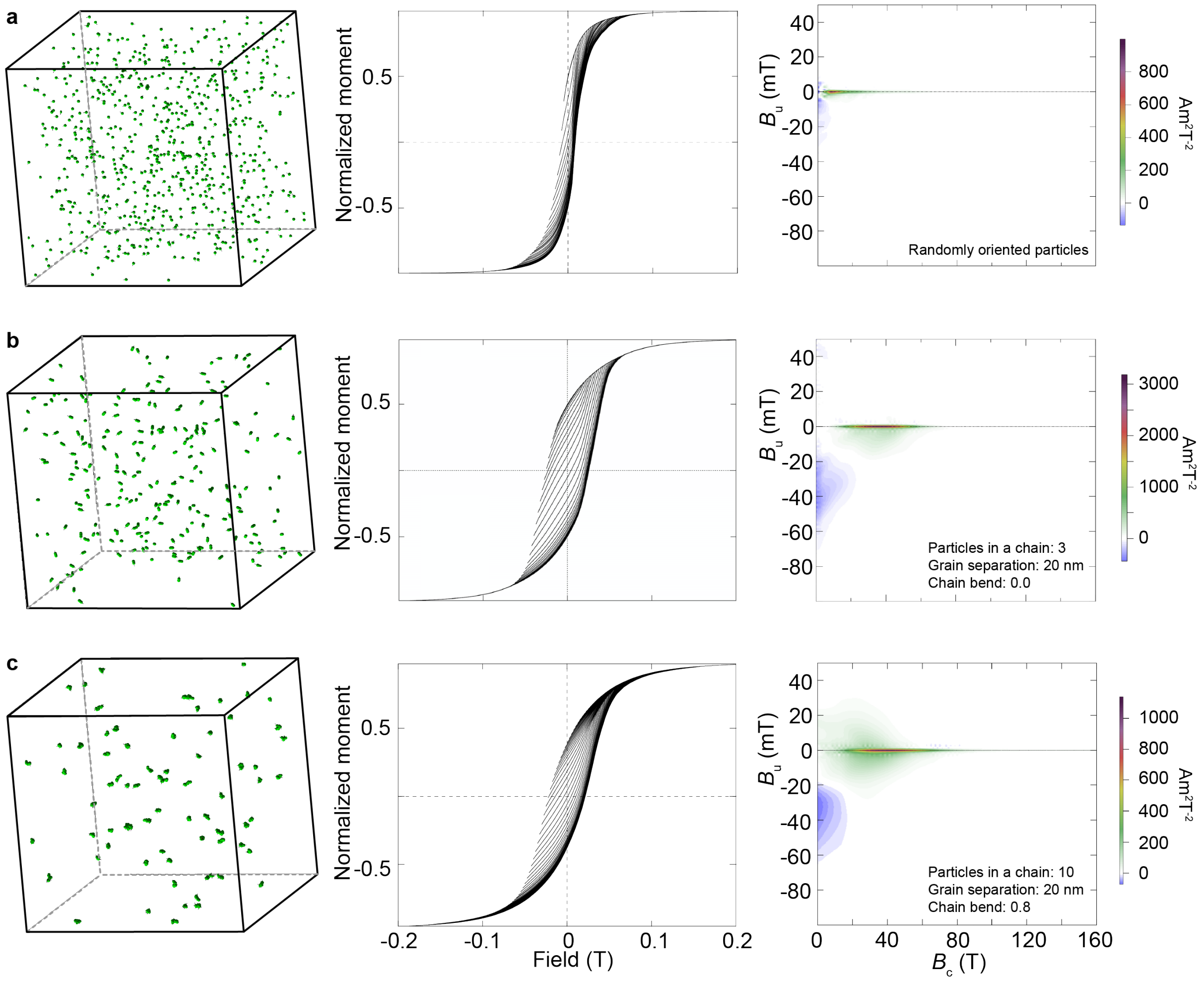

Supplementary Figure 3 Micromagnetic simulation of FORC diagrams for the PETM peak sample. The magnetofossil size distribution in the initial model (left) was constructed using TEM data from Figures 4f, i, l. Different magnetosome chain configurations (left), e.g., (a) randomly oriented, (b) 3 crystals and (c) 10 crystals in a chain, and different chain bending, were modelled. Other modelling parameters: 100 FORCs with B_c = 160 mT, B_u = 60 mT, and averaging of 100 identical FORC simulations. Simulated FORCs (middle) were processed using FORCinel[6] with VARIFORC smoothing parameters[7]: {s_{c0}, s_{c1}, s_{b0}, s_{b1}, λ_c, λ_b} = {7, 7, 2, 7, 0.1, 0.1}. Processed FORC diagrams are shown on the right. Modelling results indicate large variations in magnetic properties when different magnetic mineral microstructures are considered.

Supplementary References

1. Nowaczyk, N. R. Dissolution of titanomagnetite and sulphidization in sediments from Lake Kinneret, Israel. *Geophys. J. Int.* **187**, 34–44 (2011).
2. Roberts, A. P. Magnetic mineral diagenesis. *Earth-Sci. Rev.* **151**, 1–47 (2015).
3. Vali H. & Kirschvink, J. L. Magnetofossil dissolution in a paleomagnetically unstable deep-sea sediment. *Nature* **339**, 203–206 (1989).
4. Schumann, D., Raub, T. D., Kopp, R. E., Guerquin-Kerne, J. L., Wue, T. D., Rouiller, I., Smirnov, A. V., Kelly Sears, S., Lücken, U., Tikoo, S. M., Hesse, R., Kirschvink, J. L. & Vali, H. Gigantism in unique biogenic magnetite at the Paleocene–Eocene Thermal Maximum. *Proc. Natl. Acad. Sci. USA* **105**, 17648–17653 (2008).
5. Chang, L., Roberts, A. P., Williams, W., Fitz Gerald, J. D., Larrasoaña, J. C., Jovane, L. & Muxworthy, A. R. Giant magnetofossils and hyperthermal events. *Earth Planet. Sci. Lett.* **351–352**, 258–269 (2012).
6. Harrison, R. J. & Feinberg, J. M. FORCinel: an improved algorithm for calculating first-order reversal curve distributions using locally weighted regression smoothing. *Geochem. Geophys. Geosyst.* **9**, Q05016, doi:10.1029/2008GC001987 (2008).
7. Egli, R. VARIFORC: an optimized protocol for calculating non-regular first-order reversal curve (FORC) diagrams. *Global Planet. Change* **110**, 302–320 (2013).
